# A Case With Typical Clinical Manifestations of Kawasaki Disease and Multisystem Inflammatory Syndrome in Children Temporally Associated With SARS-CoV-2 Infection

**DOI:** 10.7759/cureus.53069

**Published:** 2024-01-27

**Authors:** Yasutaka Kuniyoshi, Hikaru Murata, Haruka Tokutake, Natsuki Takahashi

**Affiliations:** 1 Pediatrics, Tsugaruhoken Medical COOP Kensei Hospital, Hirosaki, JPN; 2 Internal Medicine, Tsugaruhoken Medical COOP Kensei Hospital, Hirosaki, JPN

**Keywords:** intravenous immunoglobulin (ivig), abdominal pain without diarrhea, sars-cov-2 infection, multisystem inflammatory syndrome in children (mis-c), kawasaki disease (kd)

## Abstract

Whether Kawasaki disease (KD) and multisystem inflammatory syndrome in children (MIS-C) temporally associated with SARS-CoV-2 infection are two distinct syndromes or part of the same spectrum is not fully understood. In this report, we present the case of a five-year-old boy who fully satisfied the diagnostic criteria for both KD and MIS-C associated with SARS-CoV-2 infection. He tested positive for SARS-CoV-2 on an oropharyngeal swab antigen test approximately four weeks before the onset of symptoms. He had severe abdominal pain. Abdominal ultrasound showed ascites. He improved with initial (2 g/kg) and additional (1 g/kg) intravenous immunoglobulin (IVIG) therapy and intravenous methylprednisolone (initial dose, 2 mg/kg/day). Our case may lead to clarification of the pathogenesis of both diseases. Additionally, the recent history of SARS-CoV-2 infection for children with prolonged fever and no clear focus of infection should be checked, and, if present, clinicians should consider MIS-C temporally associated with SARS-CoV-2 infection. IVIG therapy is important for children with MIS-C who meet the diagnostic criteria for KD, even if diagnosed with MIS-C.

## Introduction

Pediatric patients with acute coronavirus disease 2019 (COVID-19) have generally milder symptoms than adult patients [1]. However, multisystem inflammatory syndrome in children (MIS-C) temporally associated with severe acute respiratory syndrome coronavirus 2 (SARS-CoV-2), which is a serious complication and known as a pediatric inflammatory multisystem syndrome (PIMS) or pediatric inflammatory multisystem syndrome temporally associated with SARS-CoV-2 (PIMS-TS), has been reported to rarely occur in children. It was first reported in the United Kingdom [2], and then several cases were reported from COVID-19 pandemic regions. MIS-C temporally associated with SARS-CoV-2 infection has a clinical presentation similar to incomplete Kawasaki disease (KD) and toxic shock syndrome. The incidence of KD in Japan and other East Asia is high. However, reports of MIS-C following COVID-19 from East Asia areas are very few compared with those from Western countries.

The pathophysiology of MIS-C following COVID-19 is still unclear. Furthermore, whether KD and MIS-C temporally associated with SARS-CoV-2 infection are two distinct syndromes or part of the same spectrum is not fully understood. Previous studies reported that only 5% of those diagnosed with MIS-C met the diagnostic criteria for complete or typical KD [3,4].

In this report, we present the case of a Japanese boy who fully satisfied the diagnostic criteria for both KD and MIS-C temporally associated with SARS-CoV-2 infection. The possible reasons for the low incidence of MIS-C in Asian countries include the lower number of COVID-19-infected children than in Western countries and differences in genetic and ethnic backgrounds, but these are currently unknown [5]. We report this case to provide insight into the etiology of both diseases.

## Case presentation

A five-year-old previously healthy Japanese boy presented with high-grade fever for five days, abdominal pain associated with loose stools, and sore throat in September 2022. He tested positive for SARS-CoV-2 on an oropharyngeal swab antigen test approximately four weeks before the onset of symptoms. His parent also tested positive at this time. He had no past medical or family history of KD, and he had never received a COVID-19 vaccine. He also had no past history of SARS-CoV-2 infection.

**Figure 1:**
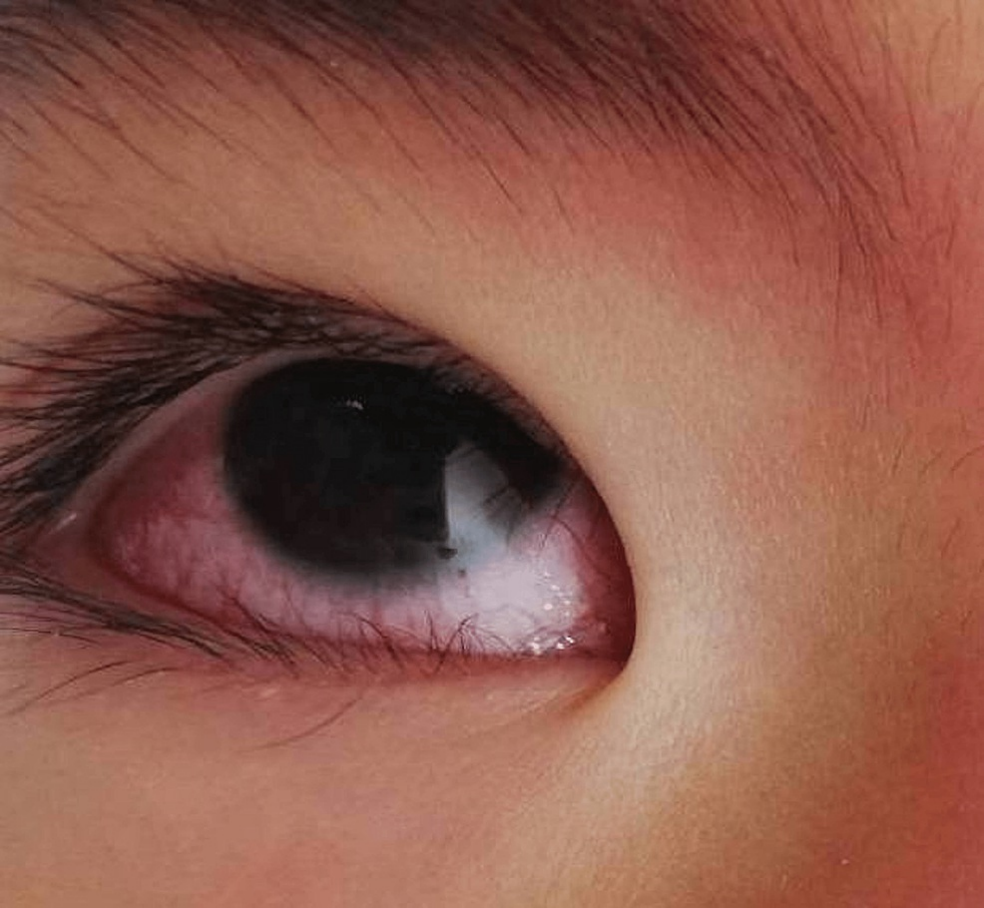
Bilateral conjunctival injection

**Figure 2:**
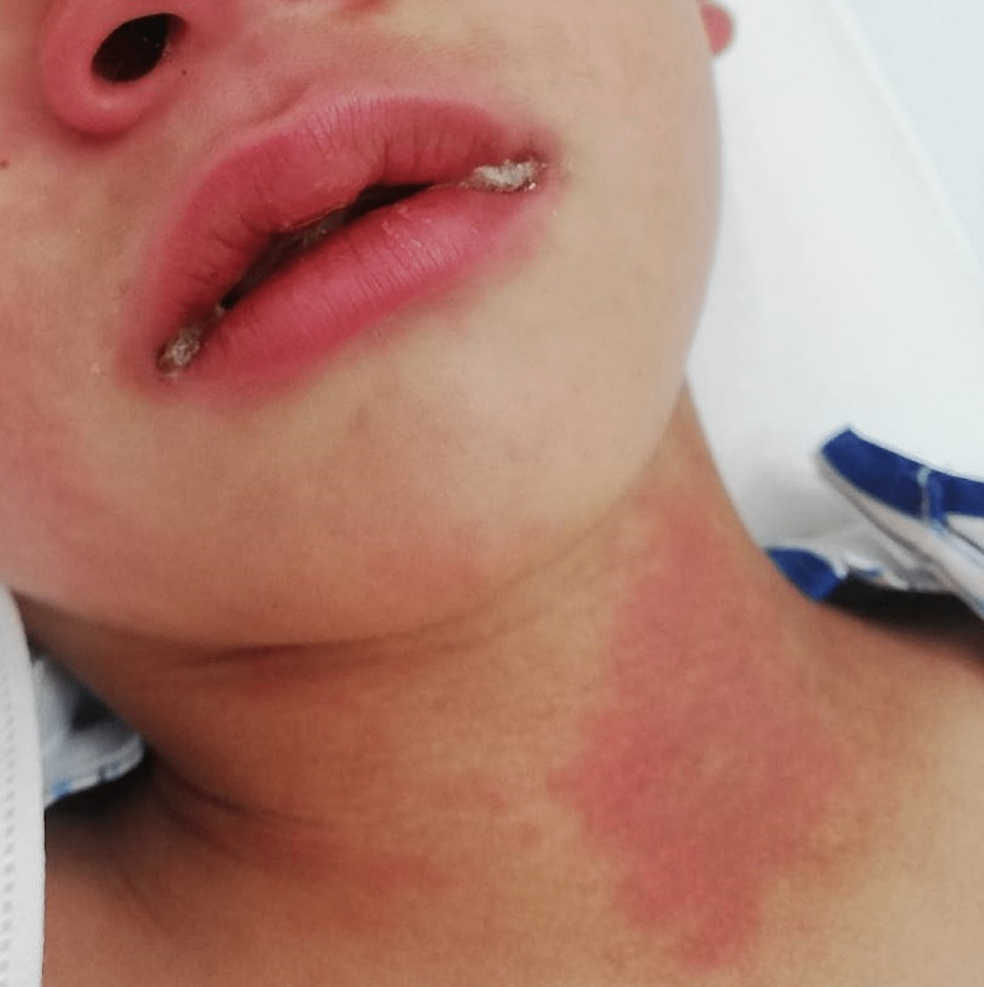
Reddening of the lips and erythema in the face and left neck

**Figure 3:**
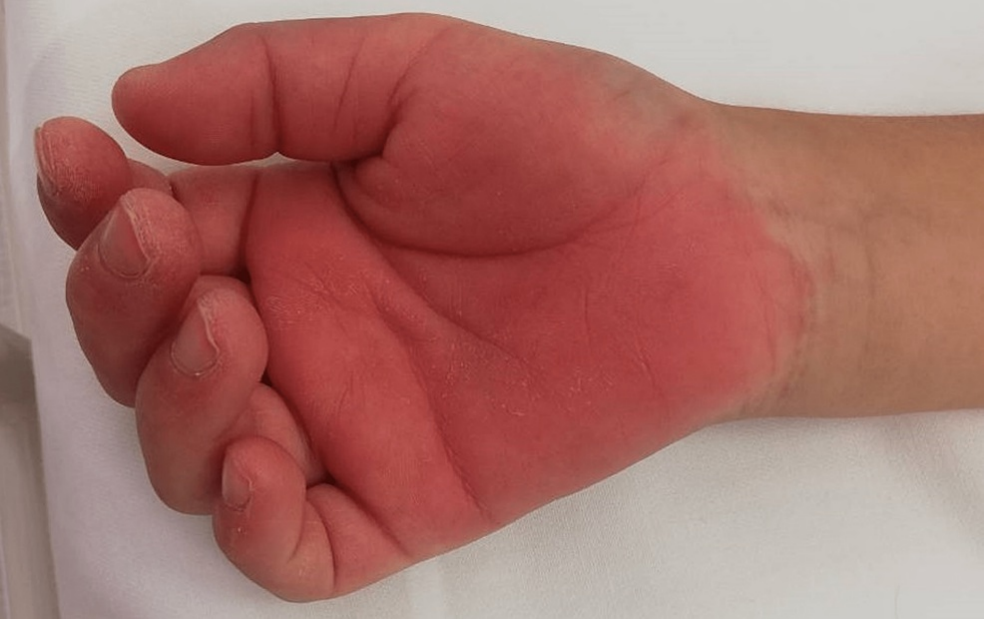
Palms with swelling and redness

On admission, his vital signs revealed high-grade fever (temperature 39.6°C), tachycardia (heart rate at 166 bpm), blood pressure of 98/60 mm Hg, respiratory rate of 28 breaths per minute, and oxygen saturation of 97% on room air. Physical examination revealed bilateral conjunctival injection without exudate (Figure 1), reddening of the lips (Figure 2), diffuse injection of oral and pharyngeal mucosae, and diffuse cervical lymphadenopathy. Lung auscultation was clear, and the heart examination was almost normal. His abdomen was soft with no organomegaly, and some tenderness throughout the abdomen was noted. His palms and soles were swelling with redness (Figure 3). He had difficulty walking due to pain caused by edema of the sole. He presented with erythema in the face, left neck, and lower back regions (Figure 2).

Initial laboratory analysis revealed leukocytosis with left shift, neutrophilia, thrombocytopenia elevated inflammatory markers (C-reactive protein of 17.21 mg/dL; ferritin of 3,690 ng/dL), elevated cardiac markers (N-terminal pro-BNP of 15,600 pg/mL), elevated liver enzymes, and hypoalbuminemia (Table 1).

**Table 1:** Laboratory analysis BNP, b-type natriuretic peptide

	Reference range	Hospital day 1	Hospital day 3	Hospital day 8	Hospital day 11
						White cell count (×10²/L)	33–90	114	117	193	198
Neutrophils (%)	34–70	90.3	76	68.7	77.7						
						Lymphocytes (%)	18–49	7.6	7.5	23.9	16.8
Hemoglobin (g/L)	13.5–17.5	12.9	10.7	10.5	10.3						
						Hematocrit (%)	39.7–52.4	35.7	31	30.3	29.1
Platelets (×10⁹/L)	140–340	126	205	438	447						
						Aspartate aminotransferase (units/L)	10–40	109	53	42	49
Alanine aminotransferase (units/L)	5–45	66	33	32	48						
						Lactate dehydrogenase (units/L)	124–222	492	311	306	263
Albumin (g/dL)	3.8–5.2	3.3	2.3	2.9	3.3						
						Bilirubin, total (mg/dL)	0.2–1.2	0.58	0.4	0.58	0.45
Creatine phosphokinase (units/L)	60–270	36	28	20	14						
						Blood urea nitrogen (mg/dL)	8–20	20.1	13.2	9	9.2
Creatinine (mg/dL)	0.61–1.04	0.36	0.23	0.27	0.31						
						C-reactive protein (mg/dL)	<0.3	17.21	8.92	1.27	0.46
Ferritin (ng/mL)	9.0–275	3690	2430	383	275						
						NT-pro-BNP (pg/mL)	<125	15600	–	–	236
% Prothrombin time		90.5	116.7	104.3	128.9						
						Activated partial thromboplastin time (seconds)	25–38	41.8	40.9	32.9	27.7
Fibrinogen (mg/dL)	200–400	557	320	198	147						

A SARS-CoV-2 RNA reverse transcriptase-polymerase chain reaction assay of nasopharyngeal swab and stool samples was negative at the time of admission. All microbiological cultures of blood, urine, and stool samples were also negative.

**Figure 4:**
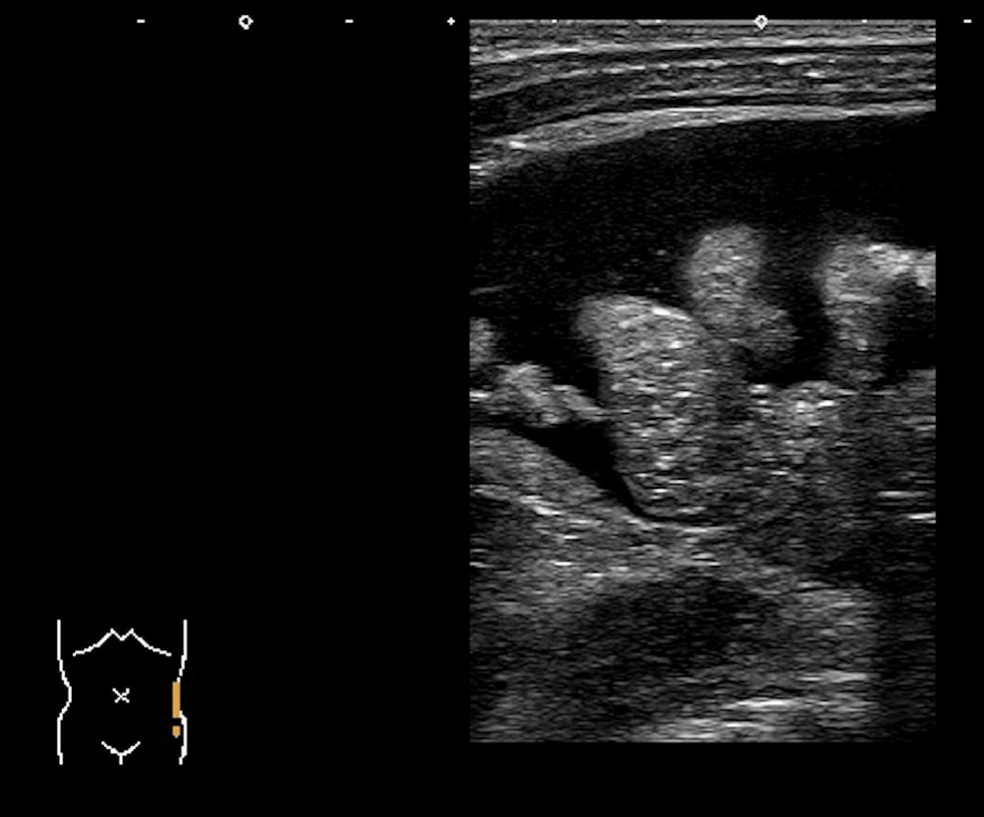
Abdominal ultrasound

**Figure 5:**
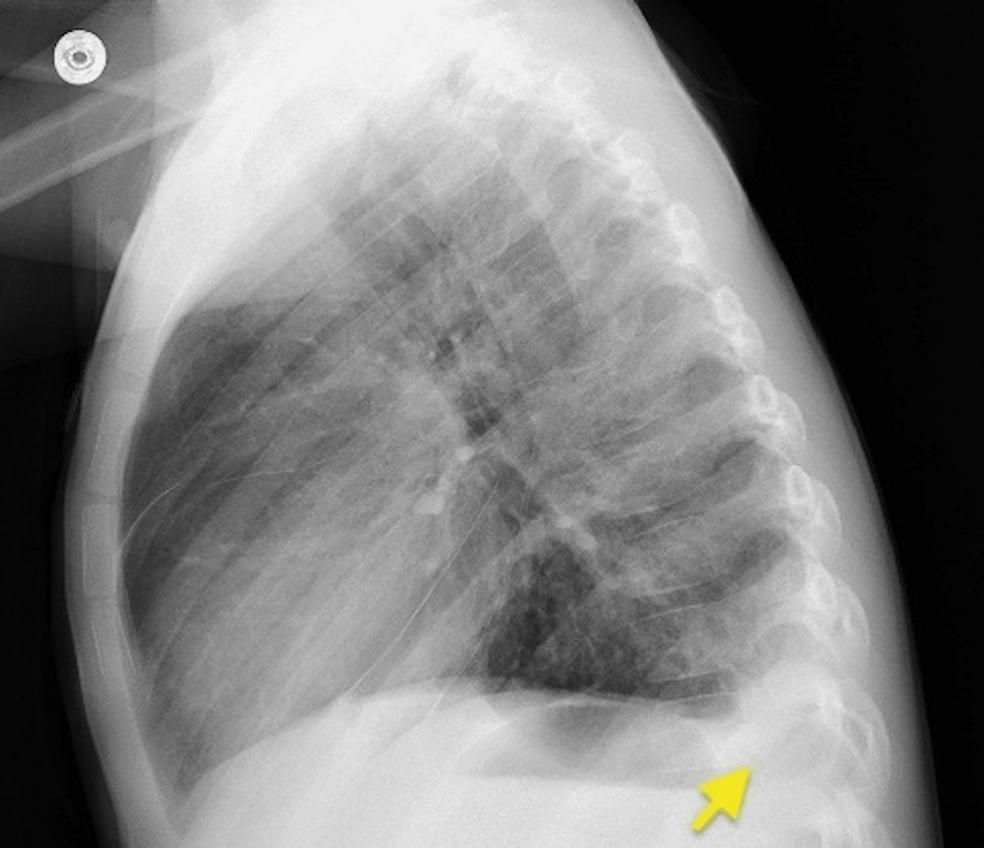
Chest radiograph

**Figure 6:**
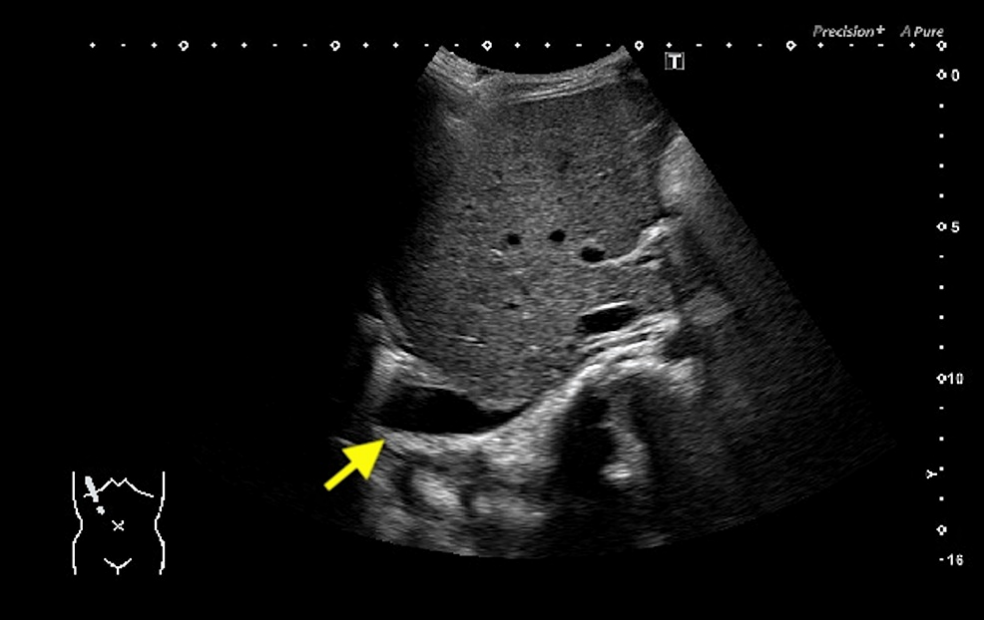
Chest ultrasound

Echocardiography showed normal left ventricular but pericardial effusion. Coronary arteries were of normal size. Abdominal ultrasound showed ascites (Figure 4). Chest radiograph (Figure 5) and ultrasound (Figure 6) showed pleural effusion.

From the time of admission, the patient was strongly suspected of KD. He met all principal clinical features for the diagnosis of KD defined by the Japanese Kawasaki Disease Research Committee and the American Heart Association (AHA) [6,7]. Additionally, he also met the MIS-C diagnostic criteria of the Centers for Disease Control and Prevention (CDC) [8], the World Health Organization (WHO) [9], and the Royal College of Paediatrics and Child Health [10].

On the first day of hospitalization (the sixth day of the illness), the patient received intravenous immunoglobulin (IVIG) 2 g/kg over 24 hours, intravenous methylprednisolone 2 mg/kg/day, and oral aspirin 10 mg/kg/day. On the second day of hospitalization, the fever resolved. On the fourth day of hospitalization, he developed a fever again, and an additional dose of IVIG (1 g/kg) over 24 hours was given. Corticosteroids were reduced to methylprednisolone 1 mg/kg/day from the fourth day of hospitalization. He was switched to oral 1 mg/kg/day of prednisone for five days from the sixth day of hospitalization and 0.5 mg/kg/day for five days from the 11th day of hospitalization. Oral famotidine (1 mg/kg/day) was administered for gastroprotection until the completion of the corticosteroid administration.

**Figure 7:**
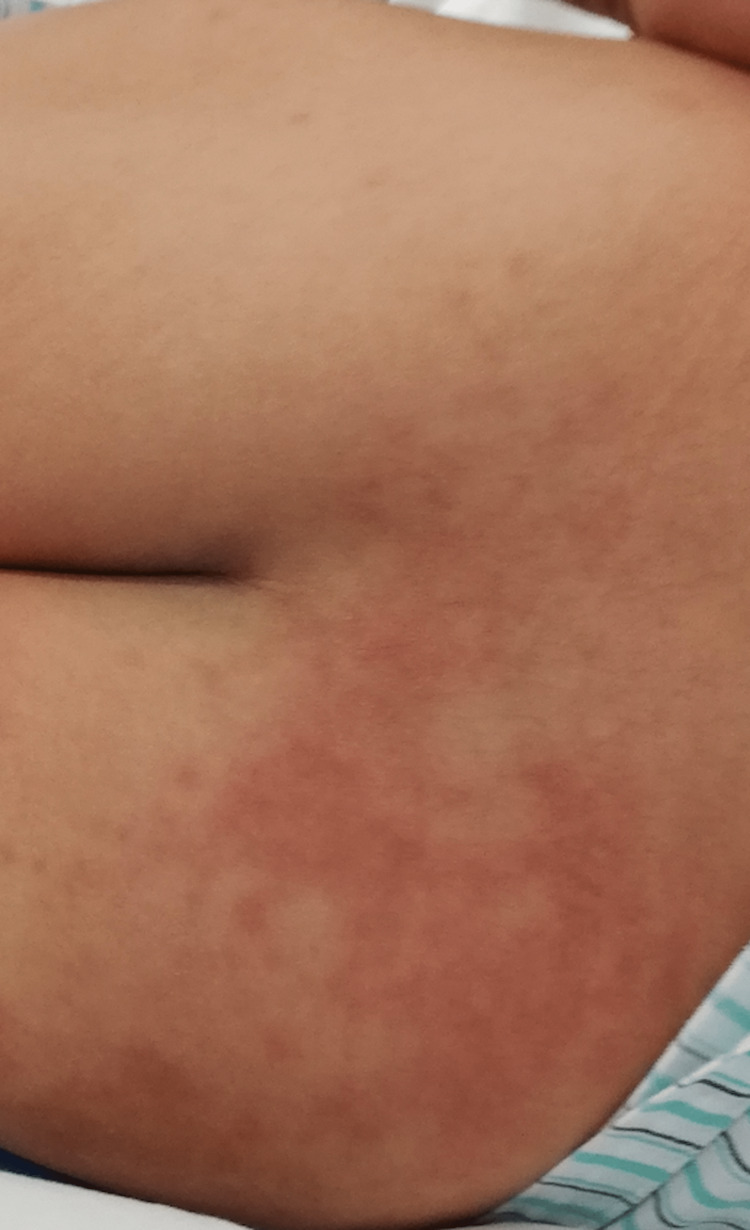
Erythema of the lower back region turned into a lightly hyperpigmented rash

**Figure 8:**
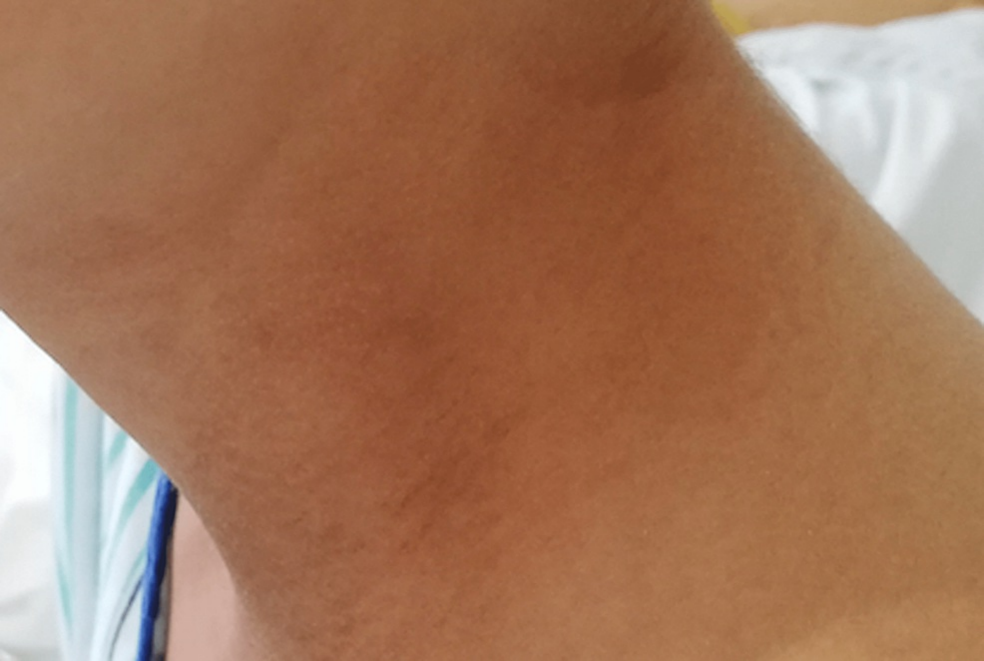
Erythema of the neck turned into a lightly hyperpigmented rash

After an additional dose of IVIG on the fourth day of hospitalization, he went down with a fever on the fifth day of hospitalization. He had severe abdominal pain that lasted until the fifth day of hospitalization and gradually disappeared. He presented with pleural effusion and ascites that gradually disappeared during the recovery phase. During hospitalization, swelling with redness was marked, and skin desquamation was seen on the entire palms. Erythema of the neck and lower back region turned into a lightly hyperpigmented rash along the course of the illness (Figures 7, 8).

He was discharged on the 16th day of hospitalization (day 21 of the illness). At discharge, he still had congestion of the capillaries of the ocular conjunctiva. Repeated follow-up echocardiography during hospitalization and at one month of onset showed normal coronary artery Z-scores and normal myocardial function.

We obtained written informed consent from the patient’s family.

## Discussion

We report the case of a Japanese boy who fully satisfied the diagnostic criteria for both KD and MIS-C following SARS-CoV-2 infection. The onset of symptoms four weeks after COVID-19 is typical for MIS-C temporally associated with SARS-CoV-2 infection.

The patient met all the diagnostic criteria for KD. He met 6/6 of the principal clinical features in the Japanese Kawasaki Disease Research Committee guidelines and 5/5 of the principal clinical features in the AHA guidelines [6,7]. The response to the initial IVIG treatment was reasonably good. Although this case corresponded to an IVIG non-responder, the fever went down after the first IVIG treatment and went down after the second IVIG treatment after recurrence. Based on these results, patients meeting the diagnostic criteria for complete or incomplete KD should be initially treated with a full dose of IVIG according to the treatment of KD, even if they meet the diagnostic criteria for MIS-C.

The fact that there was a latency of approximately four weeks from SARS-CoV-2 infection to the onset of MIS-C was similar to the results of epidemiological studies [11]. Gastrointestinal symptoms of severe abdominal pain and ascites are also typical of MIS-C [12]. Gastrointestinal symptoms are the second most common symptom in MIS-C, but they are less common in KD, at approximately 10% [13].

As the patient had typical clinical manifestations of both KD and MIS-C, it was unlikely that one had met the diagnostic criteria for the other by coincidence. A recent study using artificial intelligence to compare the gene expression signatures of KD and MIS-C suggested that the profiles of host immune responses in KD and MIS-C patients are similar [14]. It is hypothesized that KD and MIS-C are not different diseases but have overlapping features reflecting different parts of the disease spectrum [15].

In Japan, the incidence of KD was dramatically reduced during the COVID-19 pandemic in 2020 due to the decrease in various infections other than SARS-CoV-2 infection hypothetically [16,17]. However, the number of pediatric patients increased rapidly after the emergence of the Omicron variant, with more than 100 MIS-C cases being reported in Japan as of May 2023 [11]. It has been reported that receiving two doses of COVID-19 vaccination is highly effective in preventing MIS-C [18]. Therefore, COVID-19 vaccination for eligible children should be recommended.

## Conclusions

The recent history of SARS-CoV-2 infection for children with prolonged fever and no clear focus of infection should be checked, and, if present, clinicians should consider MIS-C temporally associated with COVID-19. IVIG therapy is important for children with MIS-C who meet the diagnostic criteria for KD, even if diagnosed with MIS-C. SARS-CoV-2 infection may trigger KD and MIS-C as the same spectrum.
